# Effects of neuraxial anesthesia in sitting and lateral positions on maternal hemodynamics in cesarean section: A systematic review and meta-analysis

**DOI:** 10.1371/journal.pone.0303256

**Published:** 2024-05-17

**Authors:** Cui Wen, Ying-ying Xiang, Qian-yun Pang, Hong-liang Liu

**Affiliations:** Department of Anesthesiology, Chongqing University Cancer Hospital, Chongqing, China; Kasr Alainy Medical School, Cairo University, EGYPT

## Abstract

**Study objective:**

During cesarean section, hypotension is a frequent side effect of spinal anesthesia. As a sitting or lateral position is required for spinal anesthesia performance, which of these two positions is more likely to cause intraoperative nausea, vomiting, and hypotension is still unknown. This meta-analysis compared the effects of these two positions on maternal hemodynamics and intraoperative nausea and vomiting.

**Design:**

Systematic review and meta-analysis

**Setting:**

Operating room

**Patients:**

This study included 803 patients from 12 randomized controlled trials (RCTs).

**Interventions:**

Neuraxial anesthesia in sitting position vs. lateral position.

**Measurements:**

We chose RCTs comparing the effects of spinal anesthesia in the sitting and lateral positions on maternal hemodynamics by thoroughly searching PubMed, Embase, the Cochrane Library, and the Web of Science for articles published from database inception until October 31, 2022. The Cochrane Handbook was used to assess the methodological quality of each RCT; the results were analyzed using RevMan 5.4 software; and the Egger test was used to assess publication bias.

**Main results:**

12 randomised controlled trials with 803 participants were ultimately included in the final analysis. No significant differences were observed between the two positions in terms of the incidence of hypotension(RR, 0.82; 95% CI, 0.58–1.16; *P* = 0.26; *I*^*2*^ = 66%), lowest systolic blood pressure(MD, -0.81; 95% CI, -7.38–5.75; *P* = 0.81; *I*^*2*^ = 86%), the dose of ephedrine(MD, -1.19; 95% CI, -4.91–2.52; *P* = 0.53; *I*^*2*^ = 83%), and number of parturients requiring ephedrine(RR, 0.97; 95% CI, 0.64–1.46; *P* = 0.88; *I*^*2*^ = 74%). For the incidence of intraoperative nausea and vomiting, there was no statistical difference between the two positions.

**Conclusion:**

Parturients undergoing elective cesarean section under spinal anesthesia in the sitting or lateral position experienced similar incidence of hypotension, and there were no significant differences between these two positions in terms of the amount of ephedrine administered or the number of patients needing ephedrine. In both positions, the frequency of nausea and vomiting was comparable. The ideal position for anesthesia can be chosen based on the preferences and individual circumstances of the parturient and anesthesiologist.

## Introduction

Spinal anesthesia is widely used in obstetrical and gynecological surgeries [1, 2], which can provide perfect analgesic effect and rapid recovery. In cesarean sections, spinal anesthesia is frequently utilized, which minimizes the risk of neonatal exposure to anesthesia and maternal complications from general anesthesia. However, spinal anesthesia is associated with an increase in the diffusion rate of local anesthetics as a result of the reduction in sympathetic tone brought on by the local anesthetics and the compression of the subarachnoid space by the gravid uterus. As a result, anesthesia during labor is frequently complicated by hypotension, with a 50% incidence rate [3]. Unpleasant parturient symptoms such as nausea, vomiting, dyspnea, and cerebral hypoperfusion can be induced by transient hypotension. Persistent severe hypotension may lead to a loss of consciousness, ischemia of vital organs, cardiovascular failure, and fetal acidosis due to uterine placental hypoperfusion [4]. Strategies for prevention and treatment of hypotension usually include fluid infusion, administration of vasoactive drugs, displacement of the uterus, compression of the lower extremities, and administration of low concentrations of the anesthetic. However, these strategies cannot completely prevent hypotension [5].

The sitting position is easier for puncture positioning than the lateral position [6], which is the other commonly used position for spinal anesthesia puncture. However, the parturient may have a negative experience in this position. Although the parturient may feel more at ease in the lateral position, unilateral block is more likely to occur. The impact of these two postures on maternal hemodynamic parameters is still debatable at present. Some studies reported that maternal hemodynamics are more stable in the sitting than in the lateral position [7, 8], while others showed the opposite results [9, 10]. Therefore, we conducted a systematic review and meta-analysis of randomized controlled trials (RCTs) that investigated the effects of the postural position on the hemodynamics of women undergoing spinal anesthesia. Our study compared the effects of the sitting and lateral postures on the incidence of maternal hypotension, intraoperative nausea and vomiting, lowest systolic blood pressure, amount of ephedrine used, and the number of women needing ephedrine.

## Methods

The protocol of this systematic review and meta-analysis was registered on PROSPERO(registration number:CRD42022380679) in December 2022. This meta-analysis was performed by the Preferred Reporting Items for Systematic Reviews and Meta-Analysis (PRISMA) guidelines.

### Information sources and search strategy

We meticulously searched PubMed, Embase, Cochrane library, and Web of Science databases using a combination of relevant medical subject heading (MeSH) terms and text words including sitting position, lateral position, cesarean section with the Boolean search terms ‘OR’ and ‘AND’ in accordance with the PRISMA guidelines [11] (S1 File). The databases were searched until October 31, 2022, and we also manually searched for reference lists of eligible studies and previous systematic reviews. We excluded observational studies, reviews, abstracts, case reports, conferences and letters.

### Eligibility criteria

Studies were included if the PICOS (population, intervention, comparison, outcomes, study design) criteria were met: (a) Parturients at full-term gestation and presenting for elective cesarean delivery; (b)Spinal anesthesia or combined spinal-epidural anesthesia in lateral position; (c) Spinal anesthesia or combined spinal-epidural anesthesia in sitting position; (d) Main outcomes: incidence of hypotension, lowest systolic blood pressure, dose of ephedrine, number of parturients who required ephedrine, the incidence of nausea and vomiting; (e) Included studies were limited to RCTs. This study excluded emergency cesarean sections, and articles published in languages other than English.

### Study selection and data extraction

Two researchers (CW and YYX) independently screened and assessed the titles, abstracts, and full-text articles. Full-text copies of potentially relevant articles were obtained and reviewed for eligibility. Disagreements were resolved through consensus, or if no consensus could be reached, a third researcher (QYP) provided an opinion. Two researchers (CW and YYX) extracted data independently and in duplicate using a pre-assigned standardized data summary sheet. A third reviewer (QYP) was responsible for adjudicating disagreements. We collected data on trial characteristics, demographic data, intervention and control procedures, and primary outcomes. Incomplete or missing data were requested by e-mail from the original author.

### Quality assessment

The quality of the included RCTs was assessed using the Revised Cochrane Risk of Bias 2.0 tool independently by two authors (CW and YYX). We used the tool to assess risk of bias (RoB) in the following domains: randomization process, deviations from intended interventions, missing outcome data, measurement of the outcome, and selection of the reported result. We rated each domain as ’low’, ’some concerns’ or ’high’.

### Statistical methods

All extracted data were statistically analyzed using Review Manager 5.3 software (Cochrane Collaboration, Oxford, UK). Mean difference (MD) with 95% confidence interval (CI) was used to represent continuous variables (lowest systolic blood pressure and ephedrine dose). Dichotomous variables (incidence of hypotension, number of patients requiring ephedrine, and incidence of nausea and vomiting) were analyzed using relative risk (RR) with 95% CI. The *χ*^*2*^ test and *I*^*2*^ value were used to measure heterogeneity, and heterogeneity was considered present when *I*^*2*^ >50%. The random effects model was selected for studies with heterogeneity (*P*<0.1 or *I*^*2*^ ≥50%), while the fixed effects model was selected for those without heterogeneity (*P*>0.1 or *I*^*2*^ <50%). To investigate the source of heterogeneity, subgroup analysis was used. We did not assess publication bias due to the fewer than 10 included studies for each outcome.

## Results

### Study selection

**Fig 1:**
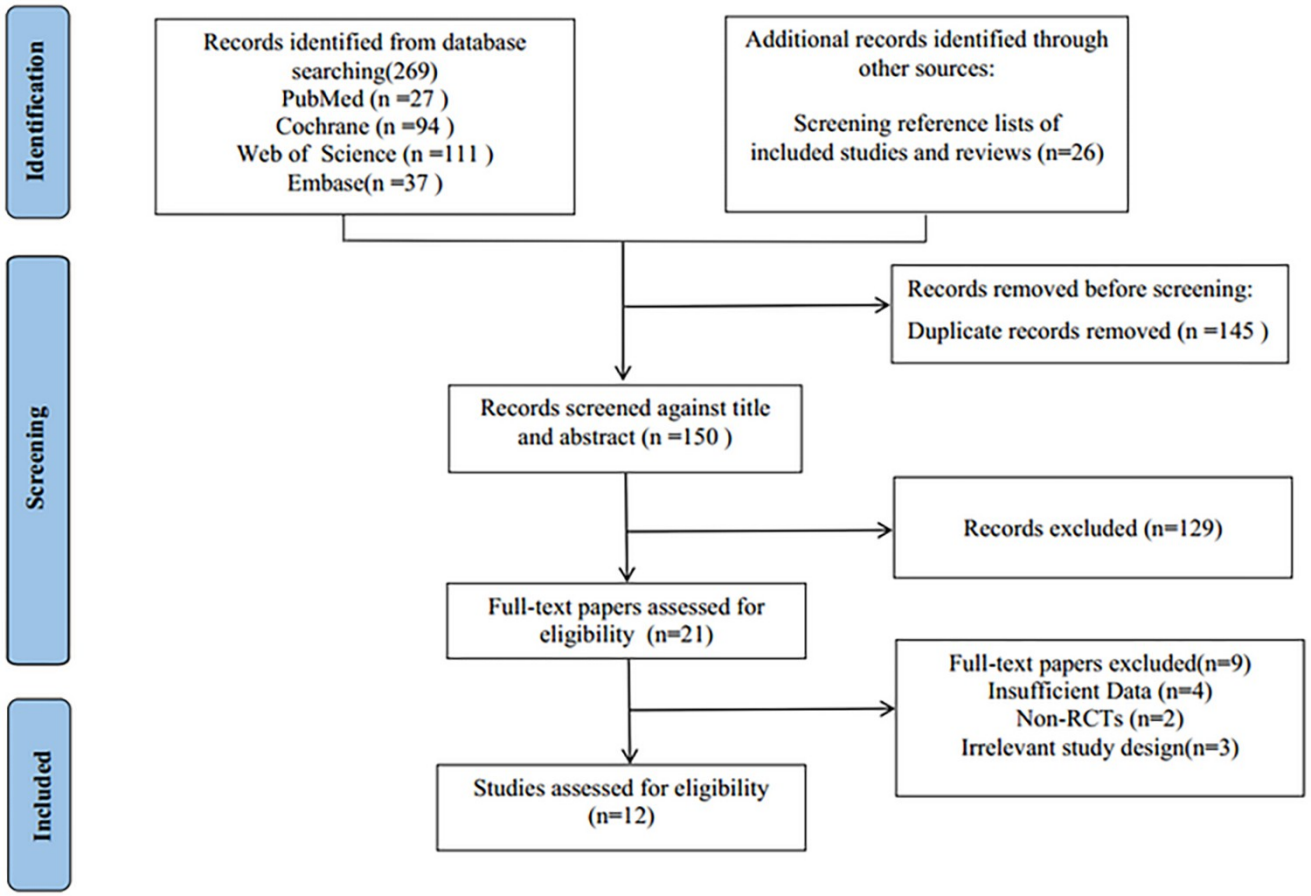
PRISMA flow chart of the meta-analysis.

We initially searched 295 articles from the database and other sources, and excluded 145 articles that were duplicates. the full version of 21 were retrieved after screening and detailed selection. Finally, 803 participants from 12 studies [7–10, 12–19] were included in this meta-analysis. The flow chart of study selection was shown in Fig 1.

### Study characteristics

The details of the study characteristics were shown in Table 1. Spinal anesthesia was performed in 5 studies [9, 12, 13, 17, 19], combined spinal-epidural anesthesia was performed in 7 studies [8, 10, 14–18]. A total of 803 participants were enrolled, 415 patients from lateral position group, 388 patients from sitting group. All of the included studies were randomly grouped.

**Table 1:** Characteristics of included studies.

Author/year	No. Total (L/S)	country	Centre	Age (L/S)	Type of anesthesia	Puncture site	Spinal anaesthesia drugs	analgesia levels	Main outcomes
Coppejans et al, [8] 2006	28/28	Belgium	Single	32±4/31±4	CSE	L_3-4_/ L_4-5_	hyperbaric bupivacaine 6.6mg+3.3 μg Sufentanil	T_6_	①②③④⑤
Inglis et al, [12] 1995	18/20	Britain	single	29/28	SA	L_2-3_	0.5% hyperbaric bupivacaine 2.5ml	T_6_ / T_4_	①②③
Manouchehrian et al, [9] 2021	53/52	Iran	single	30±6/31±5	SA	L_3-4_	0.5% hyperbaric bupivacaine (2ml) + 2.5μg sufentanil	T_6_	①②③④⑤
Obasuyi et al, [13] 2013	50/50	Africa	single	31±4/32±4	SA	L_3-4_	0.5% plain bupivacaine 2ml or 2.4ml	T_6_	①③④⑤
Okucu et al, [14] 2021	50/50	Turkey	single	31±4.8/31.7±4.7	CSE	L_2-3_ / L_3-4_	0.5% isobaric bupivacaine 1.8 ml	T_6_	②④⑤⑦⑥
Patel et al, [15] 1993	24/24	London	single	33.9/34.3	CSE	L_2-3_	0.5% hyperbaric bupivacaine 2ml	T_4_	①③④⑤⑥
Russell et al, [16] 2002	30/30	UK	Single	33±3.4/33.5±4.9	CSE	L_3-4_	0.5% hyperbaric bupivacaine 2.5ml + 12.5 μg fentanyl	T_5_	③④⑤⑥
Simin et al, [7] 2018	38/38	Palestine	Single	28.7±5.8/30.8±5.5	SA	L_3-4_ / L_4-5_	0.5% hyperbaric bupivacaine 2ml +15μg fentanyl	T_4_	①④⑤⑥
Tan et al, [17] 2014	30/30	Turkey	Single	32±4.6/32.1±4.5	CSE	L_3-4_ / L_3-4_	0.5% heavy bupivacaine 2ml +20 μg fentanyl	T_6_	①③④⑥
Xu et al, [18] 2016	59/29	China	Single	30±4/31±4	CSE	L_3-4_	0.5% hypobaric ropivacaine 2.5ml	T_6_	①③④⑤⑥
Yun et al, [10] 1998	10/12	America	Single	33.1±2.8/33.6±4.5	CSE	L_2-3_ / L_3-4_	0.75% hyperbaric bupivacaine 12mg + 10 μg fentanyl	T_4_	①②③④⑤
Prakash et al, [19] 2013	25/25	India	single	26.6±4.2/25±2.7	SA	L_3-4_	0.5% hyperbaric bupivacaine 2ml	T_5_	③④⑥

CSE: combined spinal with epidural; SA: spinal anesthesia; ①Hemodynamics, ②Vasopressor doses, ③Block characteristics, ④ Apgar score, ⑤Nausea/vomiting, ⑥Vasopressor requirements.

### Risk of bias summary

**Fig 2:**
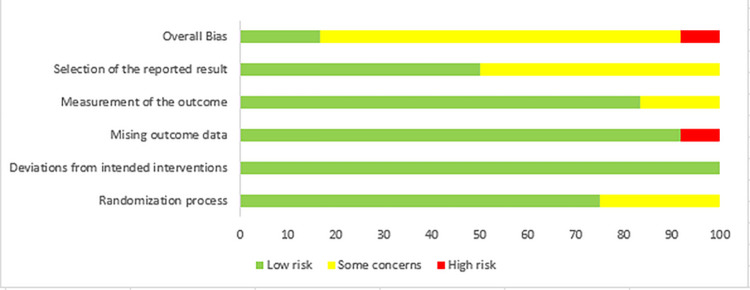
Risk of bias assessment using the cochrane risk of bias 2.0 tool.

**Fig 3:**
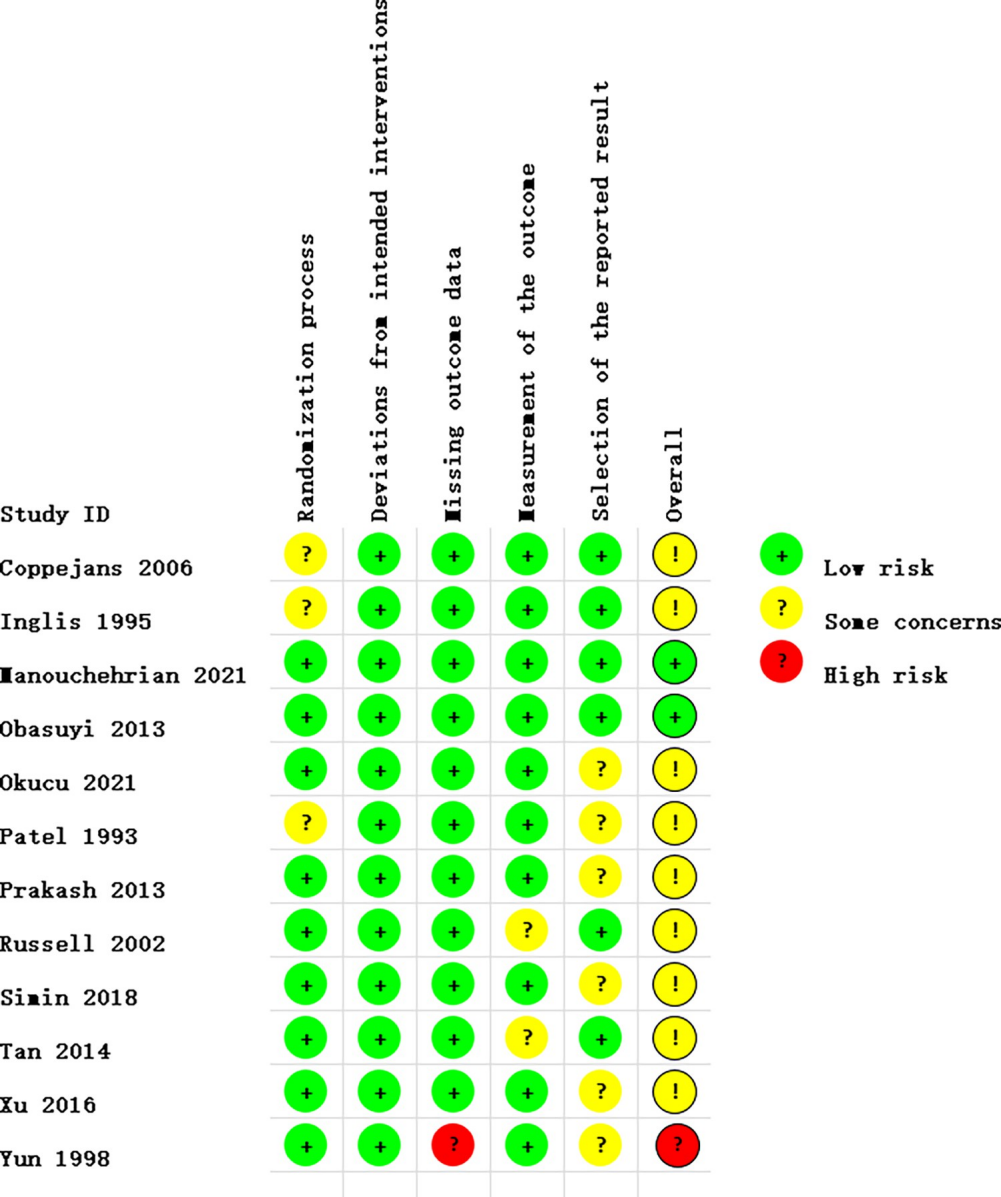
Risk of bias assessments on the five domains of each study.

The quality of the included RCTs was assessed using the Revised Cochrane Risk of Bias 2.0 tool [20], The risk of bias of the included studies was shown in Fig 2. From the Cochrane Risk of Bias assessment, 2 RCTs [9, 13] were classified as having a low risk, 9 RCTs [7, 8, 12, 14–16, 17–19]were classified as having ‘some concerns’, and 1study [10] was classified as having a high risk. Fig 3 summaries the risk of bias assessments on the five domains of each study.

### Incidence of hypotension

**Fig 4:**
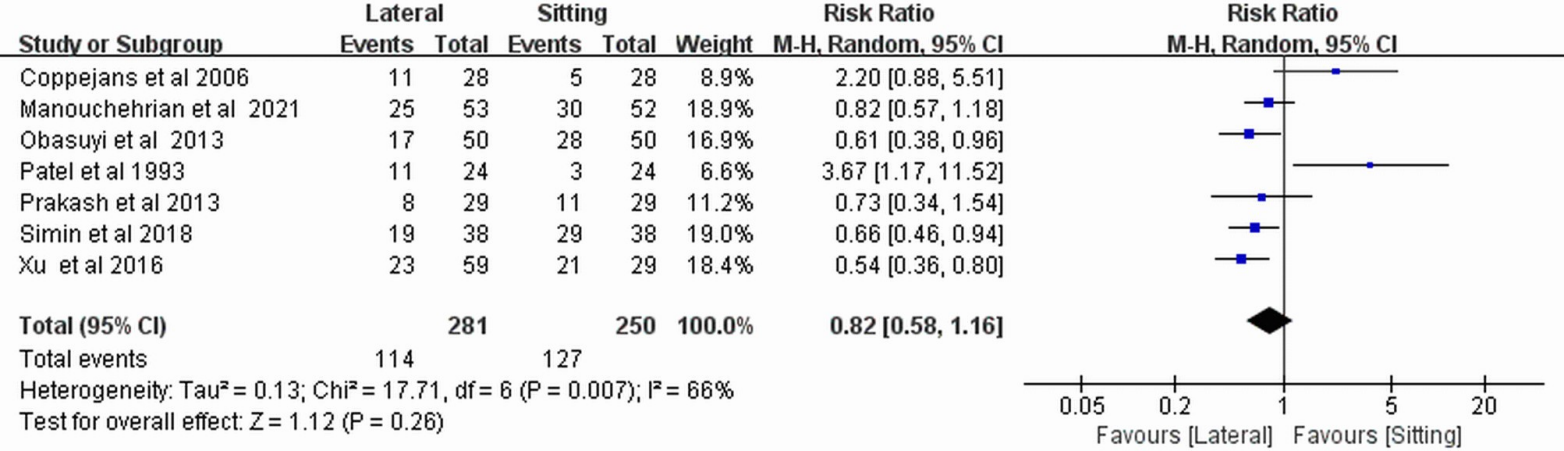
Forest plot of the incidence of hypotension.

In both the sitting and lateral positions, maternal hypotension was observed in seven trials [7–9, 13, 15, 18, 19], and there was no between-group difference (RR, 0.82; 95% CI, 0.58–1.16; *P* = 0.26; *I*^*2*^ = 66%) (Fig 4). Subgroup analysis showed that there was no significant difference between the two groups for the incidence of hypotension when bupivacaine was used alone (RR, 1.03; 95% CI, 0.42–2.51; *P* = 0.95; *I*^*2*^ = 77%) or when bupivacaine combined with opioid analgesics used (RR, 0.9; 95% CI, 0.55–1.46; *P* = 0.66; *I*^*2*^ = 67%) (S1 Fig). The meta-analytic results of the effect of the two postures on the incidence of hypotension were robust according to the results of the sensitivity analysis (S2 Fig).

### Lowest systolic blood pressure

**Fig 5:**
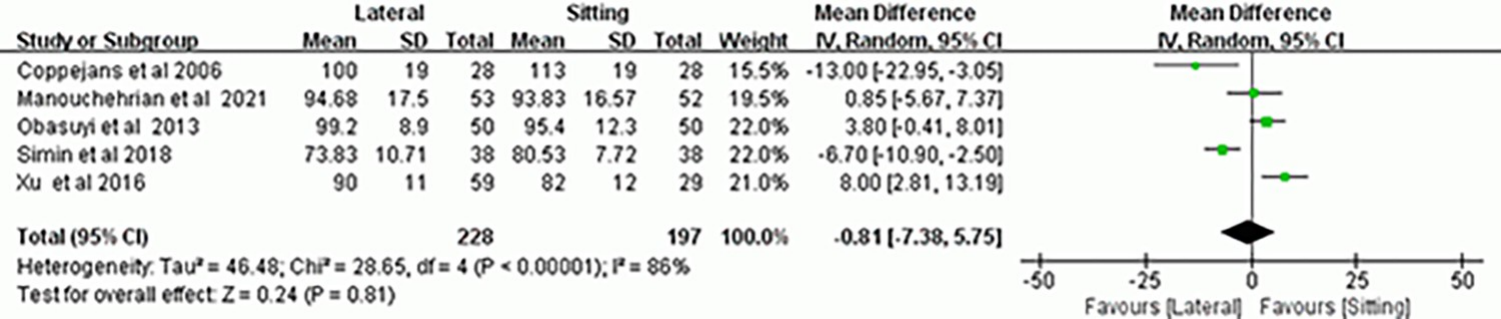
Forest plot of meta-analysis of lowest systolic blood pressure.

The results of five RCTs [7–9, 13, 18] comparing the maternal lowest systolic blood pressure following spinal anesthesia in the two positions did not differ statistically (MD, -0.81; 95% CI, -7.38–5.75; *P* = 0.81; *I*^*2*^ = 86%) (Fig 5). The results of the sensitivity analysis revealed that the meta-analytic findings on the effect of the two anesthetic positions on the the lowest blood pressure values were robust (S3 Fig).

### Dose of ephedrine

**Fig 6:**
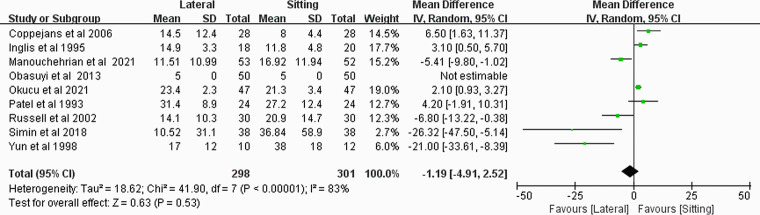
Forest plot of total dose of ephedrine (mg).

Nine RCTs [7–10, 12–16] reported the use of ephedrine in the two positions, and there was no significant difference between the two positions (MD, -1.19; 95% CI, -4.91–2.52; *P* = 0.53; *I*^*2*^ = 83%) (Fig 6). Subgroup analysis revealed that the dose of ephedrine in sitting position group was much lower than that in lateral position group when bupivacaine was used alone (MD, 2.33; 95% CI, 1.27–3.38; *P*<0.0001; *I*^*2*^ = 0%), but it did not differ between groups when bupivacaine combined with opioid analgesics used(MD, -7.69; 95% CI, -16.52–1.14; *P* = 0.09; *I*^*2*^ = 86%) (S4 Fig). The sensitivity analysis showed that, the result was not changed when omitting any study (S5 Fig).

### Number of parturients who required ephedrine

**Fig 7:**
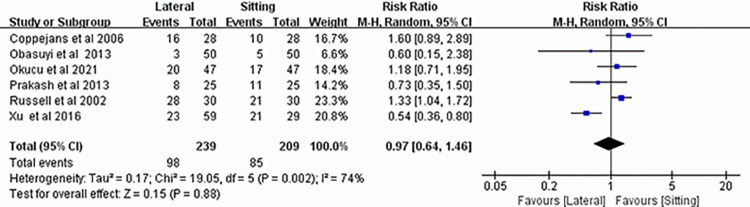
Forest plot of ephedrine requirement.

The number of parturients who needed vasopressor support following spinal anesthesia in the two positions was recorded in 6 studies [8, 13, 14, 16, 18, 19], and the results showed no statistically significant difference (RR, 0.97; 95% CI, 0.64–1.46; *P* = 0.88; *I*^*2*^ = 74%) (Fig 7). Subgroup analysis revealed that the number of parturients who required ephedrine rescue did not differ between the two groups when bupivacaine was used alone (RR, 0.96; 95% CI, 0.65–1.43; *P* = 0.85; *I*^*2*^ = 0%), but it was significant higher in the lateral position group when bupivacaine combined with opioid analgesics used. (RR, 1.37; 95% CI, 1.09–1.73; *P* = 0.008; *I*^*2*^ = 0%) (S6 Fig). The sensitivity analysis showed that, the result was not changed when omitting any study (S7 Fig).

### Incidence of nausea and vomiting

**Fig 8:**
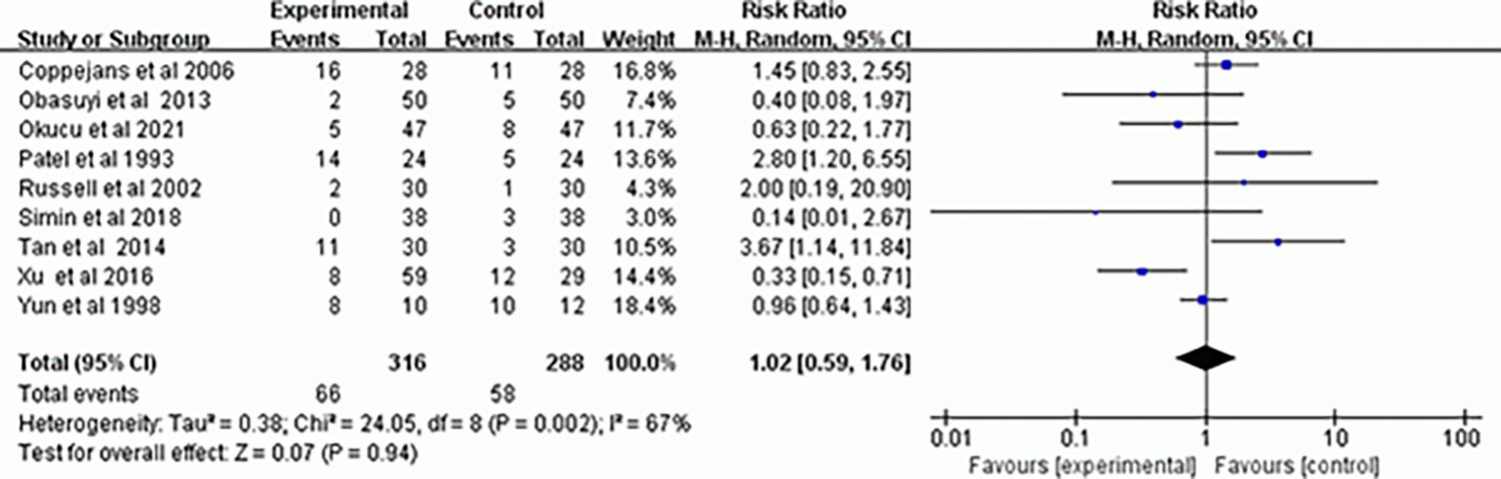
Forest plot of incidence of nausea and vomiting.

The incidence of nausea and vomiting in the two positions was compared in 9 trials [7, 8, 10, 13–18], and the findings revealed no statistically significant difference (RR, 1.02; 95% CI, 0.59–1.76; *P* = 0.94; *I*^*2*^ = 67%) (Fig 8). Subgroup analysis showed no difference in the incidence of nausea and vomiting between the groups when bupivacaine used alone (RR, 0.99; 95% CI, 0.29–3.38; *P* = 0.99; *I*^*2*^ = 72%) or when bupivacaine combined with opioid analgesics used (RR, 1.34; 95% CI, 0.74–2.6; *P* = 0.34; *I*^*2*^ = 52%) (S8 Fig).The results of the sensitivity analysis on the incidence of nausea and vomiting in the two positions were robust (S9 Fig).

## Discussion

To our knowledge, this meta-analysis is the first to assess how the administration of spinal anesthesia in the sitting and lateral positions affects maternal hemodynamics as well as intraoperative nausea and vomiting. The two positions were evaluated in a total of 12 RCTs with 803 participants. According to the meta-analytic findings, there was no statistically significant difference between the two positions in terms of the incidence of hypotension and nausea and vomiting, lowest systolic blood pressure, amount of ephedrine administered, and number of parturients requiring ephedrine during elective cesarean section.

### Incidence of hypotension and lowest systolic blood pressure

After spinal anesthesia, the sympathetic block brought on by the rapid cephalad diffusion of the local anesthetic may contribute to maternal hypotension. Different body positions may affect maternal hemodynamics because the posture of the parturient affects how quickly the local anesthetics diffuse in the cerebrospinal fluid [21]. The sitting and lateral positions are the two commonly used positions for spinal anesthesia. Studies have shown that in the sitting position, the hyperbaric local anesthetic spreads more caudally due to the effect of gravity [22], while in the lateral position it spreads more cephaladly [23]. So, the level of block is higher in the lateral position when hyperbaric local anesthetics are used, and the likelihood of hypotension is increased. Coppejans et al [8] found that during spinal anesthesia with hyperbaric bupivacaine, parturients in the lateral position had a higher level of block and a higher incidence of hypotension compared to those in sitting position. However, Yun et al [10] discovered that parturients in the sitting position experienced a more sustained decline in blood pressure. According to a study by Hwang et al [21], the hemodynamics of parturients were more stable, and the dose of vasoactive medications was lower in the lateral position. Our meta-analysis found no statistically significant difference between the two positions with regard to the incidence of maternal hypotension and the lowest systolic blood pressure.

Intrathecal opioids can enhance the block from spinal anesthesia. The subgroup analysis in our meta-analysis showed that there was no significant difference between the sitting and lateral positions when bupivacaine was used alone or bupivacaine used combined with opioids. We thought the heterogeneity mainly came from the differences in the interval of blood pressure monitoring, the time of epidural catheter insertion, the speed of administration, the dose of local anesthetics, and other factors. For example, Obasuyi et al [13] recorded the blood pressure every 1 min for the first 10 min, every 3 min for the next 20 min, and every 5 min thereafter. However, blood pressure was taken by Okucu et al [14] every minute from the start of anesthesia until delivery and then every 5 min until the end of the procedure. In addition, there is no accepted definition of hypotension following spinal anesthesia for cesarean section. Fifteen distinct definitions of hypotension was reported by Klöhr et al [24], the different various criteria for hypotension may be a cause of heterogeneity as well.

### Dose of ephedrine and number of parturients requiring it

The use of a vasoactive agent is the main prevention and treatment strategy for hypotension after spinal anesthesia [25]. Ephedrine is the preferred vasoactive agent in obstetric anesthesia. According to the meta-analyses published in 2002 by Lee et al [26] and Lin et al [27], parturients who were given ephedrine showed lower pH values in their newborns’ cord blood. Therefore, it is important to identify ways to decrease the prevalence of maternal hypotension and use of ephedrine. Some researchers have studied the use of ephedrine in parturients after spinal anesthesia in different positions. According to Russell et al [16], the amount of ephedrine required in parturients following the administration of hyperbaric bupivacaine in the sitting spinal anesthesia position was higher than when it was given in the lateral position. However, Tan et al. [17] reported that the ephedrine dose in parturients was similar in the two positions. Our meta-analysis found no statistically significant difference in ephedrine requirement between the two positions. According to the subgroup analysis, the dose of ephedrine was considerably lower in parturients anesthetized in the sitting position compared to the lateral position when bupivacaine was alone used. This is consistent with the findings of a study by Inglis [12] and may be caused by the higher level of block in the lateral position, which may lead to a more significant drop in blood pressure and a greater need for ephedrine. The number of parturients who required ephedrine was higher in the lateral position group and the ephedrine dose was similar in both groups when using bupivacaine combined with opioids. Intrathecal opioids may reduce the need for local anesthetics and lessen the degree of sympathetic blockade, which may affect the ephedrine dose in lateral position. Further research is required to verify these findings due to the individual differences in the maternal response to ephedrine and the varying standards for hypotension used in trials.

### Nausea and vomiting

Intraoperative nausea and vomiting is a common complication in parturients after spinal anesthesia [28]. Although hypotension is the primary causative factor, other potential causes include vagal hyperactivity, visceral pain, and intravenous opioid supplementation. According to previous studies, methods for treating hypotension are less likely to alleviate nausea and vomiting than those for preventing it [29]. Study have shown that the incidence of nausea and vomiting in parturients under anesthesia in the lateral position was higher than that in the sitting position [5]. This may be due to a higher prevalence of nausea and vomiting brought on by the secondary maternal hypotension induced by the fast sympathetic blockade in the lateral than in the sitting position. Our meta-analysis explored the effect of spinal anesthesia on the severity of hypotension in two different positions in parturients and also compared its effect on intraoperative nausea and vomiting, the differences were not statistically significant.

Our meta-analysis has several limitations. First, the included participants were all parturients who had elective cesarean sections, and there was a lack of comparison with the emergency cases. Second, the included RCTs were not double-blinded. Third, large heterogeneity existed in the included studies. Fourth, the sample size of included studies were small, only 803 participants from 12 studies were included, especially for the subgroup analyses. Multicenter RCTs with large sample sizes are needed in the future.

## Conclusion

In summary, our meta-analysis shows that the incidence of hypotension, the lowest systolic blood pressure, dose of ephedrine used, and number of parturients who required ephedrine were similar between patients undergoing elective cesarean section and receiving spinal anesthesia in the sitting or lateral positions. There was also no difference in the incidence of nausea and vomiting between the two positions. The appropriate position can be selected according to the preference of the parturient and anesthesiologist, or the physical condition of the parturient. Further research is required to verify these findings due to the high heterogeneity and small sample sizes of the included studies.

## Supporting information

S1 Checklist PRISMA 2020 checklist. (DOCX)

S1 File Search strategy. (PDF)

S1 Fig Subgroup analysis of incidence of hypotension. (TIF)

S2 Fig The sensitivity analysis of incidence of hypotension. (TIF)

S3 Fig The sensitivity analysis of lowest systolic blood pressure. (TIF)

S4 Fig Subgroup analysis of dosage of ephedrine. (TIF)

S5 Fig The sensitivity analysis of dosage of ephedrine. (TIF)

S6 Fig Subgroup analysis of number of parturients who required ephedrine. (TIF)

S7 Fig The sensitivity analysis of number of parturients who required ephedrine. (TIF)

S8 Fig Subgroup analysis of incidence of vomiting and nausea. (TIF)

S9 Fig The sensitivity analysis of incidence of vomiting and nausea. (TIF)
