# Supplementary material for: Effects of neuraxial anesthesia in sitting and lateral positions on maternal hemodynamics in cesarean section: A systematic review and meta-analysis
Source: PLoS One. 2024 May 17;19(5):e0303256. doi: 10.1371/journal.pone.0303256 (PMC11101069; doi:10.1371/journal.pone.0303256)
Supplement: S1 File — (PDF) [file pone.0303256.s002.pdf]

Search strategy for WOS

No.	Search Query	Results
#1	sitting position (Topic) OR Sitting Positions (Topic) OR Sitting (Topic) OR Seated Position (Topic) OR Seated Positions (Topic) OR Position, Seated (Topic) OR Positions, Seated (Topic) and Preprint Citation Index (Exclude-Database)	887800
#2	lateral position (Topic) and Preprint Citation Index (Exclude-Database)	194513
#3	Cesarean Section (Topic) OR Cesarean Sections (Topic) OR Delivery, Abdominal (Topic) OR Abdominal Deliveries (Topic) OR Deliveries, Abdominal (Topic) OR Caesarean Section (Topic) OR Abdominal Delivery (Topic) OR Caesarean Sections (Topic) OR C-Section (Topic) OR C Section (Topic) OR C-Sections (Topic) OR Postcesarean Section (Topic) and Preprint Citation Index (Exclude-Database)	575523
#4	#1 AND #2 AND #3 and Preprint Citation Index (Exclude-Database)	111

Search strategy for PubMed

No.	Query	Results
#9	(("Cesarean Section"[Mesh]) OR ((((((Cesarean Section[Title/Abstract]) OR (Abdominal Deliveries[Title/Abstract])) OR (Caesarean Section[Title/Abstract])) OR (Abdominal Delivery[Title/Abstract])) OR (C-Section[Title/Abstract])) OR (Postcesarean Section[Title/Abstract]))) AND ((("Sitting Position"[Mesh]) OR (((sitting position[Title/Abstract]) OR (Seated Position[Title/Abstract])) OR (Sitting[Title/Abstract]))) AND (lateral position[Title/Abstract]))	27
#8	("Cesarean Section"[Mesh]) OR ((((((Cesarean Section[Title/Abstract]) OR (Abdominal Deliveries[Title/Abstract])) OR (Caesarean Section[Title/Abstract])) OR (Abdominal Delivery[Title/Abstract])) OR (C-Section[Title/Abstract])) OR (Postcesarean Section[Title/Abstract]))	76,883
#7	(((((Cesarean Section[Title/Abstract]) OR (Abdominal Deliveries[Title/Abstract])) OR (Caesarean Section[Title/Abstract])) OR (Abdominal Delivery[Title/Abstract])) OR (C-Section[Title/Abstract])) OR (Postcesarean Section[Title/Abstract])	53,314
#6	"Cesarean Section"[Mesh]	54,313
#5	(("Sitting Position"[Mesh]) OR (((sitting position[Title/Abstract]) OR (Seated Position[Title/Abstract])) OR (Sitting[Title/Abstract]))) AND (lateral position[Title/Abstract])	175
#4	lateral position[Title/Abstract]	3,493
#3	("Sitting Position"[Mesh]) OR (((sitting position[Title/Abstract]) OR (Seated Position[Title/Abstract])) OR (Sitting[Title/Abstract]))	29,719
#2	((sitting position[Title/Abstract]) OR (Seated Position[Title/Abstract])) OR (Sitting[Title/Abstract])	29,429
#1	"Sitting Position"[Mesh]	1,614

Search strategy for Embase

No.	Query	Results
#10	#3 AND #6 AND #9	37
#9	#7 OR #8	147913

#8	birth, abdominal operation':ab,ti OR 'birth, caesarean':ab,ti OR 'caesarean birth':ab,ti OR 'caesarean section':ab,ti OR 'caesarian birth':ab,ti OR 'cesarean delivery':ab,ti OR 'cesarian section':ab,ti OR fetectomy:ab,ti OR 'repeated cesarotomy':ab,ti OR 'sectio caesarea':ab,ti OR 'cesarean section':ab,ti	91994
#7	'cesarean section'/exp	134946
#6	#4 OR #5	5297
#5	'lateral position':ab,ti	5258
#4	'lateral position'/exp	142
#3	#1 OR #2	28582
#2	('posture, sitting':ab,ti OR 'sedentary activity':ab,ti OR sitting:ab,ti) AND 'activity, sedentary':ab,ti	161
#1	'sitting'/exp	28490

Search strategy for Cochrane

No.	Query	Results
#12	#6 AND #11	94
#11	#7 OR #8 OR #9 OR #10	18588
#10	(C-Sections):ti.ab.kw OR (Postcesarean Section):ti.ab.kw	625
#9	(Caesarean Section):ti,ab,kw OR (Caesarean Sections):ti,ab, kw OR (Abdominal Delivery):ti,ab,kw OR (C-Section):ti,ab,kw OR (C Section):ti,ab, kw	18562
#8	("cesarean section"):ti, ab, kw OR (Cesarean Sections):ti, ab, kw OR (Delivery, Abdominal):ti, ab, kw OR (Abdominal Deliveries):ti,ab,kw OR (Deliveries, Abdominal):ti,ab,kw	16681
#7	MeSH descriptor: [Cesarean Section] explode all trees	4492
#6	#4 AND #5	715
#5	lateral position	4827
#4	#1 OR #2 OR #3	11694
#3	(Position, Seated):ti, ab, kw OR (Positions, Seated):ti, ab, kw OR (Seated Positions):ti,ab, kw	1221
#2	(sitting position):ti, ab, kw OR (Position, Sitting):ti,ab, kw OR (Sitting Positions):ti, ab, kw OR (Sitting):ti, ab, kw OR (Seated Position):ti, ab, kw	11647
#1	MeSH descriptor: [Sitting Position] explode all trees	270
