# Supplementary figures and images for: Effects of neuraxial anesthesia in sitting and lateral positions on maternal hemodynamics in cesarean section: A systematic review and meta-analysis
Source: PLoS One. 2024 May 17;19(5):e0303256. doi: 10.1371/journal.pone.0303256 (PMC11101069; doi:10.1371/journal.pone.0303256)

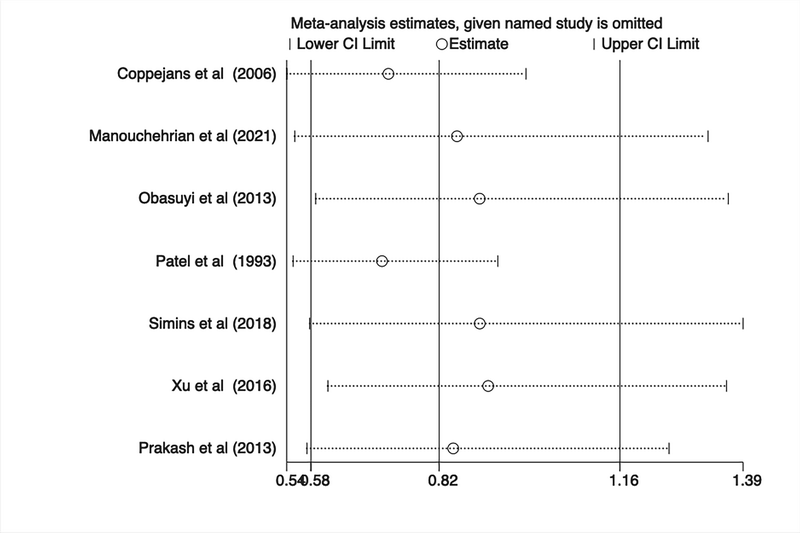

Supplement: S2 Fig — (TIF) [file pone.0303256.s004.tif]

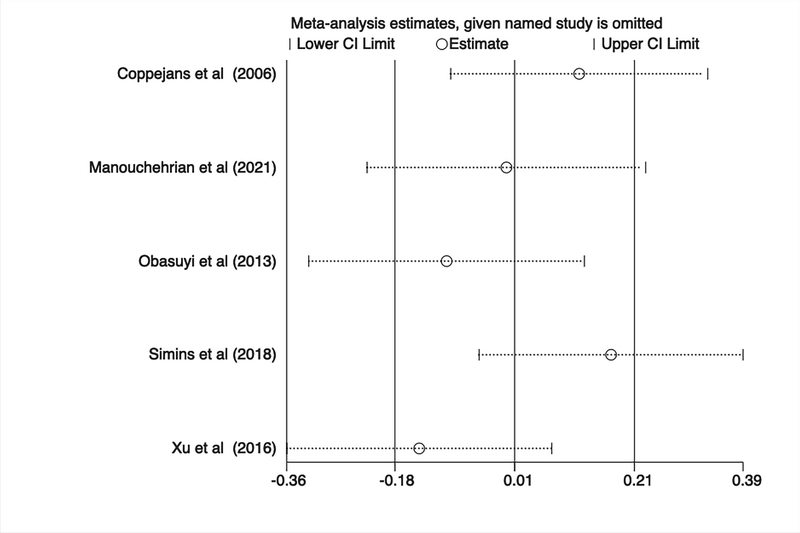

Supplement: S3 Fig — (TIF) [file pone.0303256.s005.tif]

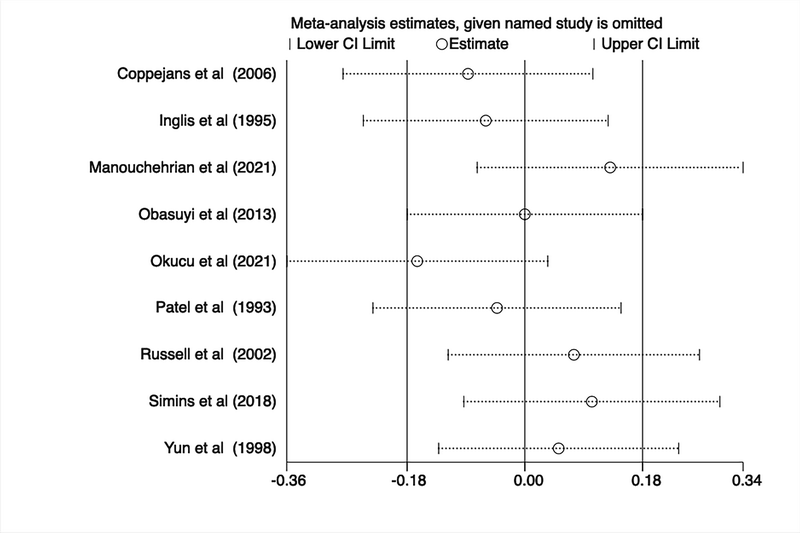

Supplement: S5 Fig — (TIF) [file pone.0303256.s007.tif]

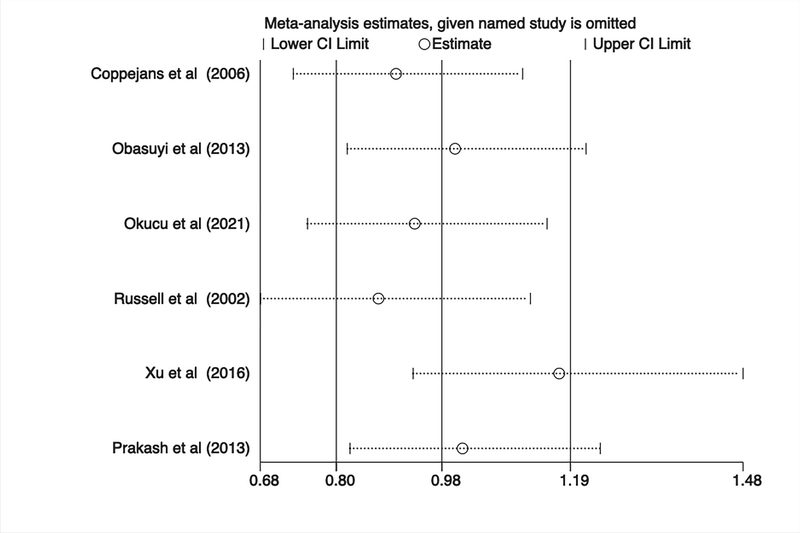

Supplement: S7 Fig — (TIF) [file pone.0303256.s009.tif]

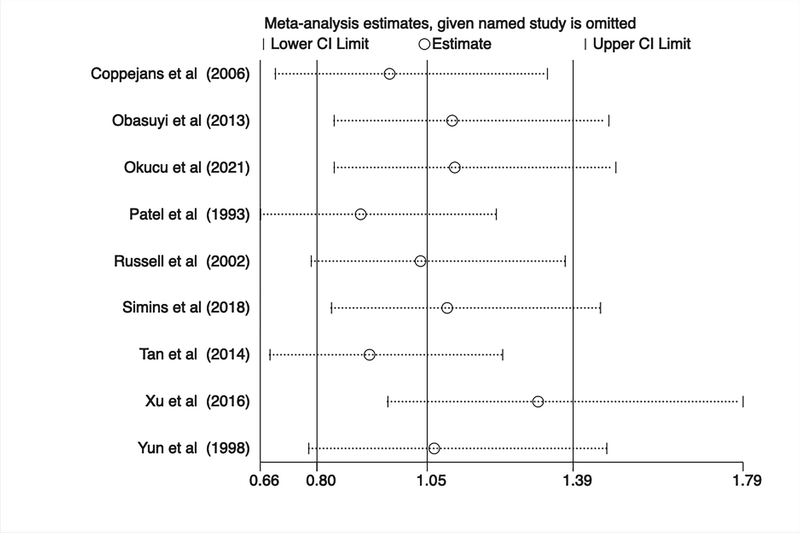

Supplement: S9 Fig — (TIF) [file pone.0303256.s011.tif]
